# Qualitative research in gerontology: rigor, legacy, and the next wave of innovation

**DOI:** 10.1093/geroni/igaf140

**Published:** 2025-12-13

**Authors:** Sean N Halpin, Tracie C Harrison, Angie K Perone, Natalie D Pope, Abby J Schwartz

**Affiliations:** RTI Health Solutions, Research Triangle Park, North Carolina, United States; College of Nursing, University of Iowa, Iowa City, Iowa, United States; School of Social Welfare, University of California, Berkeley, Berkeley, California, United States; College of Social Work, University of Kentucky, Lexington, Kentucky, United States; School of Social Work, East Carolina University, Greenville, North Carolina, United States

As editorial board members for *Innovation in Aging* specializing in qualitative methods, we see tremendous potential for the journal to become a hub for methodologically rigorous and innovative qualitative research. Yet qualitative studies remain underrepresented and, when submitted, sometimes fall short of the standards that would position them for the journal’s highest impact (Putnam, 2025). Too often, qualitative research is dismissed as anecdotal or untrustworthy, a perception the field can counter through renewed commitment to rigor, reflexivity, and transparency. In this Viewpoints article, we call for work that advances qualitative methods alongside empirical findings and builds on gerontology’s long tradition of methodological innovation to establish *Innovation in Aging* as a home for the next wave of qualitative research.

## The rigor imperative

Critiques of qualitative research are familiar. Data are rarely shared, raising concerns about fabrication; quotes are selectively chosen, suggesting cherry-picking; and subjectivity fuels mistrust among quantitatively oriented audiences. Others question whether researchers impose their own assumptions or theoretical lens on participants’ accounts or note insufficient transparency in analytic decisions and reporting.

These concerns span epistemological traditions and fuel ongoing debates about what constitutes rigor in qualitative research. Positivists may worry about validity and reliability, while constructivists emphasize reflexivity and transparency. Yet the most rigorous qualitative studies do not resolve these critiques by mirroring quantitative standards. Qualitative methods stand on their own as credible forms of inquiry. They are not preliminary to quantitative validation, nor do they require claims of generalizability to demonstrate rigor.

Rigor in qualitative research emerges through transparency, epistemological alignment, and reflexive engagement. Strong manuscripts make analytic reasoning visible, link methods to underlying assumptions, and acknowledge how interpretation is shaped rather than hidden. They demonstrate alignment between design and epistemology, present a clear analytic logic, and articulate how findings advance conceptual or applied understanding in aging. Table 1 summarizes common challenges we observe in qualitative submissions and the indicators of rigor and innovation that highlight exemplary work.

**Table 1.** Common pitfalls in qualitative submissions vs. indicators of rigor and innovation.

Pitfalls	Example	Indicators of rigor & innovation
**Mislabelling methods**	Calling descriptive coding “grounded theory.”	Clear articulation of method and rationale for its use; demonstration of analytic logic (e.g., constant comparison in grounded theory).
**Weak or absent epistemological alignment**	Methods, questions, and assumptions do not fit together.	Coherence between epistemology, research aims and chosen methods; clarity on how assumptions inform design and interpretation, grounded in established methodological literature with transparent justification for any adaptations or variations.
**Vague or absent sampling rationale**	“We used convenience sampling” with no justification.	Transparent description and justification of sampling approach, with attention to diversity, saturation, or longitudinal follow-up where relevant.
**Opaque analytic process**	Thematic claims presented without explaining how data support them.	Description of how analytic decisions were made, including how codes and themes were developed, patterns interpreted, and findings linked to research questions and conceptual framing; supported audit trails, coding frameworks, and reflexive acknowledgment of researcher role.
**Findings purely descriptive**	Findings presented as surface-level themes with no conceptual contribution.	Results linked to conceptual, theoretical, or practice implications; evidence interpretations were built through attention to nuance, including negative cases.
**Selective or “illustrative” quotes only**	Only one quote used to “prove” a theme.	Multiple excerpts showing breadth, depth, and nuance.
**Participant bias unaddressed**	No discussion of recall limitations or social desirability in interviews.	Discussion of data limitations; triangulation (caregiver + participant; observation + interview) to bolster credibility.

## The field of gerontology’s legacy of innovation

Gerontology has long been a source of methodological creativity and development in shaping qualitative research. For example, Life Review originated as a framework for understanding reminiscence in later life (Butler, 1963) and has since informed therapeutic practice in palliative care, trauma recovery, and developmental psychology, demonstrating innovations from aging research can transcend disciplines. Grounded Theory, first used in studies of social processes around dying in hospital settings (Glaser & Strauss, 1965, 1967), aligned closely with gerontology and now permeates qualitative inquiry. Likewise, Qualitative Longitudinal Research was refined within aging studies to capture lived experience over time (Cole, 1984); it now anchors studies of youth transitions, health trajectories, and policy change. The field of gerontology has proven it can contribute creative and rigorous methodological approaches that are applicable across disciplines. Now is the time to reclaim that leadership to drive the next iteration of qualitative innovation.

## The next wave

Today’s research landscape presents both urgency and opportunity for qualitative inquiry in gerontology. Questions of dementia inclusion, caregiving across cultures, and the digital lives of older adults demand approaches that are methodologically inventive and ethically attuned. The next wave of qualitative research in gerontology will be driven not from replication but from re-imagination, by developing methods that meet these challenges head-on.

For example, netnography offers tools for examining how aging identities are formed, negotiated, and resisted in online spaces (Kozinets, 2020). Photovoice, a participatory arts-based approach, facilitates the co-creation of knowledge with older adults and empowers participants to shape research narratives (Novek et al., 2012). Other participatory and arts-based methods enable co-creation of knowledge with older adults, moving them from passive participants to partners (Parti et al., 2026). Another example is Artificial Intelligence (AI)-assisted analysis, which presents the possibility of scaling qualitative inquiry while preserving interpretative depth (Hitch, 2024).

For this *Innovation in Aging editorial board*, the next step is to champion manuscripts that do more than apply qualitative methods; they should advance them. Authors should clearly identify what is innovative about their approach and what is translatable, how their methodological contribution can inform future research, practice, or policy. Doing so will move the journal beyond showcasing qualitative studies to cultivating a laboratory for methodological progress.

## Conclusion

Qualitative research in gerontology has generated methodological innovations that shaped entire disciplines. To remain credible and relevant, the field must recommit to transparency, epistemological alignment, and methodological creativity. The next era of qualitative research in aging will depend not on defending its legitimacy but on demonstrating its indispensability. By publishing work that exemplifies rigor and innovation, *Innovation in Aging* can lead this charge, showcasing studies that earn trust, push conceptual boundaries, and reaffirm qualitative inquiry as central to understanding the complexities of aging.

## Funding

None declared.

## Conflict of interest

None declared.

## Data Availability

No data were used for this viewpoint article. As such, there are no data to share. The work was not preregistered.
